# Maize *ZmRPH1* encodes a microtubule‐associated protein that controls plant and ear height

**DOI:** 10.1111/pbi.13292

**Published:** 2019-12-03

**Authors:** Wei Li, Fanghui Ge, Zhiquan Qiang, Lei Zhu, Shuaisong Zhang, Limei Chen, Xiqing Wang, Jiansheng Li, Ying Fu

**Affiliations:** ^1^ State Key Laboratory of Plant Physiology and Biochemistry College of Biological Sciences China Agricultural University Beijing China; ^2^ National Maize Improvement Centre of China China Agricultural University Beijing China

**Keywords:** maize, plant height, *ZmRPH1*, microtubule‐associated protein, yield

In grass crops, short stature is an ideal agronomic trait since it allows for better resistance to lodging and higher planting density. Dwarf and semi‐dwarf mutants have been widely used in rice (*Oryza sativa*) and wheat (*Triticum aestivum*) breeding, and the related ‘Green Revolution’ genes, such as *sd1* in rice and *Rht* in wheat, are usually involved in the gibberellin (GA) biosynthesis or signalling (Peng *et al.*, 1999; Sasaki *et al.*, 2002). However, many GA biosynthesis/signalling deficient maize (*Zea mays*) mutants have pleiotropic phenotypes that are detrimental to yields (Chen *et al.*, 2014; Fujioka *et al.*, 1988; Lawit *et al.*, 2010; Winkler and Helentjaris, 1995), therefore, are not applicable to maize breeding. Considering that maize plant height largely results from stem elongation driven by cell division and cell expansion within the internodes, identifying new regulators that directly regulate cell elongation may be a solution.

The microtubule plays an essential role in guiding plant cell polar growth, which is dependent on its organization and dynamics and regulated by a suite of diverse microtubule‐associated proteins (MAPs) (Chen *et al.*, 2016). However, MAPs in maize are largely unknown. Recently, several proteins of *Arabidopsis* QWRF family were identified as MAPs (Lee *et al.*, 2017; Pignocchi *et al.*, 2009). By searching the Gramene database, we revealed a gene with ID Zm00001d028073 that encoded a QWRF homolog protein in maize. We cloned the CDS of this gene into an overexpression vector, pBCXUN, driven by the maize *UBI* promoter, and introduced it into the maize inbred line B73‐329 using *Agrobacterium*‐mediated transformation. The resulting overexpressed lines (*OE2* and *OE4*) had significantly shorter and wider mesocotyls (Figure 1a–c) with parenchyma cells showing reduced length but increased width compared to B73‐329 (Figure 1d–f). We renamed this gene *ZmRPH1* (*Reducing Plant Height 1*). Furthermore, *ZmRPH1* overexpression also led to shorter roots with reduced cell length in maize seedlings (Figure 1g,h). These data suggest a general role for *ZmRPH1* in the control of polar cell growth in maize.

**Figure 1:**
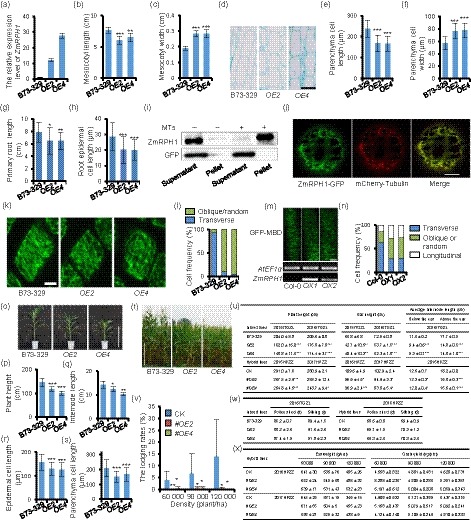
ZmRPH1 is a maize MAP that regulates plant and ear height. (a)^¶^ *ZmRPH1* expression levels analysed by RT‐qPCR in different maize lines. Internal reference: *Actin1*. (b)^¶^ Average length and (c)^¶^ width of 7‐day‐old etiolated maize mesocotyls. *n* ≥ 10. (d) Longitudinal sections of 7‐day‐old etiolated mesocotyls. Bar = 100 µm. (e)^¶^ Average length and (f)^¶^ width of mesocotyl cells shown in (d). *n* ≥ 200 cells from 10 seedlings. (g)^¶^ Average primary root length of 3‐day‐old dark‐grown maize seedlings. *n* ≥ 10. (h)^¶^ Average length of root epidermal cells in the elongation zone of different seedlings. *n* ≥ 200 cells from five seedlings. (i) Biotinylated‐lysine‐labelled ZmRPH1 but not GFP (control) cosedimented with prepolymerized microtubules in vitro. (j) ZmRPH1‐GFP colocalized with mCherry‐tubulin‐labelled microtubules in maize leaf protoplasts. Bar = 7 µm. (k) Cortical microtubules in root epidermal cells at the elongation zone from different 2‐day‐old dark‐grown maize seedlings. Bar = 5 µm. (l) Percentage of cells with a different microtubule orientation as shown in (k). *n* ≥ 200 cells from five seedlings. (m) GFP‐MBD‐labelled cortical microtubules in the hypocotyl epidermal cells of 3‐day‐old etiolated *Arabidopsis* seedlings. Lower panel: *ZmRPH1* transcripts in Col‐0, *OX1* and *OX2* seedlings analysed by RT‐PCR. Bar = 10 µm. (n) Percentage of cells with different microtubule orientation shown in (m). *n* ≥ 100 cells from 20 seedlings. (o) B73‐329 and *OEs* plants grown in a greenhouse for 8 weeks. Bar = 1 m. (p)^¶^ Average plant height of plants shown in (o). (*n* = 20). (q)^¶^ Average length of the sixth internode from plants shown in (o). (*n* = 5). (r)^¶^ Average length of epidermal and (s)^¶^ parenchyma cells in the middle of the sixth internode. *n* ≥ 200 cells from 10 plants. (t) B73‐329 and *OEs* plants grown in the field for 3 months. Bar = 1 m. (u)^¶Ɨ^ Table of plant and ear height and average internode length collected from inbred and hybrid plants grown in the field. One plot = 42 plants; *n* = 3 replicate plots. (v)^¶§Ɨ^ Lodging rate of hybrid plants (milk ripening stage) grown under different densities. (w)^¶Ɨ^ Table of flowering times (days) of inbred and hybrid plants. One plot = 42 plants; *n* = 3 replicate plots. (x)^¶§Ɨ^ Table of ear weight and grain yield per plot of hybrid plants grown at different densities. ^¶^Data are shown as the means ± SD; *P < 0.05, **P < 0.01, ***P < 0.001, *t*‐test. ^§^One plot of different densities (60 000/90 000/120 000 plants/ha) = 42/63/84 plants. *n* = 3 replicate plots. ^Ɨ^Inbred plants: year 2015/2016, Gongzhuling; Hybrid plants (CK: T13/B73‐329, *#OE*: T13/*OE*): year 2016/2017, Zhuozhou.

To test whether ZmRPH1 is a novel MAP just like its *Arabidopsis* homologs, we performed an in vitro microtubule cosedimentation assay. We adopted the TNT quick coupled transcription/translation system (Promega, Madison, WI, USA) to express the ZmRPH1 protein. Biotinylated‐lysine‐labelled ZmPRH1 protein cosedimented with prepolymerized microtubules in the pellet after a high‐speed centrifugation, indicating its direct association with microtubules in vitro (Figure 1i). We then fused ZmRPH1 to green fluorescent protein (GFP) and transiently expressed it into maize protoplasts. We observed filament‐like structures that colocalized with mCherry‐tubulin‐labelled microtubules (Figure 1j). Thus, we conclude that ZmRPH1 is an MAP in maize.

Next, we investigated the function of ZmRPH1 in microtubules. It is well established that in fast elongating cells cortical microtubules organize into a transverse parallel array to guide microfibril deposition in the cell wall. When cell growth slows or ceases, cortical microtubules reorganize into randomly or longitudinally oriented arrays (Hamada, 2014). We surveyed root epidermal cells in the elongation zone, within which the microtubules were easily visualized by immunofluorescence microscopy using an anti‐α‐tubulin antibody. In 2‐day‐old seedlings, we observed transversely oriented microtubules in 93% of the B73‐329 root epidermal cells, while <10% of *OE2* or *OE4* cells had transverse microtubules. By contrast, in most *OE2* or *OE4* cells microtubules were obliquely or randomly oriented (Figure 1k,l). We further overexpressed *ZmRPH1* in *Arabidopsis*, and the resulting overexpressing lines (*OX1* and *OX2*) had shorter etiolated hypocotyls compared with the control. By crossing *OX1* and *OX2* with a transgenic line expressing *GFP‐MBD*, we observed GFP‐MBD‐labelled microtubules in live cells. We found that *ZmRPH1* overexpression significantly reduced the frequency of cells with transverse microtubules in *OX1* and *OX2* hypocotyls (approximately 30%) compared to the control (>60%; Figure 1m,n). Overall, the above results indicated that *ZmRPH1* modulates cell elongation by regulating the cortical microtubule orientation.

We then measured plant height and the length of the sixth internode in maize plants grown in a greenhouse. The *ZmRPH1* overexpressing lines (*OE2* and *OE4*) all resulted in shorter plant heights and reduced internode lengths (Figure 1o–q). Reduced internode epidermal and parenchyma cell lengths were also revealed in *OE2* and *OE4* compared with the control, B73‐329 (Figure 1r,s), indicating that *ZmRPH1* overexpression reduced internode cell elongation and affected maize plant height. However, it remains an interesting question whether ZmRPH1, as a MAP, can also regulate mitotic microtubule organization and affect cell division. Next, we evaluated various growth‐related traits of *OE2* and *OE4* plants in field trials at multiple sites across different years. In addition to the *ZmRPH1 OE* inbred lines, we also used the T13 inbred line as the female tester to make hybrids with *OE2* and *OE4*, and the resulting hybrid lines were named #*OE*s. We found that both plant and ear heights were significantly reduced in all tested *ZmRPH1* overexpressing inbred and hybrid lines compared with corresponding controls (Figure 1t,u). Additionally, the number of internodes did not differ between transgenic lines and controls. However, average internode lengths were significantly shorter in *ZmRPH1* overexpressing plants (Figure 1u). Moreover, the lodging rate of lines *#OE2* and *#OE4* was significantly lower compared with the control (Figure 1v). Thus, *ZmRPH1* overexpression reduced plant and ear height and enhanced the lodging resistance of plants, which could be a primary precondition for efforts to achieve higher yielding maize.

Plant height is highly related to maize yield. Therefore, we explored whether *ZmRPH1* overexpression would affect maize yield and found that *ZmRPH1* overexpression had no obvious impact on flowering time and fertility (Figure 1w). We then measured the ear weight per plant and grain yield per hybrid line plot in different years and found no significant difference between *ZmRPH1* overexpression lines and the control in most cases (Figure 1x).

In summary, we identified ZmRPH1 as a novel MAP that regulates cell elongation in maize. We demonstrated that *ZmRPH1* overexpression lowered plant and ear heights by reducing the length of all internodes and increased lodging resistance, without significantly reducing maize yield. This study also proved that modulation of microtubules to control cell elongation may be a potential strategy in breeding cultivars for compact planting, and genes for MAPs in maize including *ZmRPH1* could serve as new plant height genes with practical potential in creating maize of ideal plant type.

## Author contributions

W.L., J‐S. L., X‐Q. W., and Y.F. contributed to project design. W.L., F‐H. G., Z‐Q. Q., L.Z., S‐S. Z., and L‐M. C. performed the experiments and data analysis. W.L. and Y.F. wrote the manuscript. Y.F., J‐S.L. and X‐Q.W. revised the article.

## Conflict of interest

No conflict of interests to declare.

## References

[pbi13292-bib-0001] Chen, Y. , Hou, M. , Liu, L. , Wu, S. , Shen, Y. , Ishiyama, K. , Kobayashi, M. *et al* (2014) The maize *DWARF1* encodes a gibberellin 3‐oxidase and is dual localized to the nucleus and cytosol. Plant Physiol. 166, 2028–2039. 25341533 10.1104/pp.114.247486 PMC4256885

[pbi13292-bib-0002] Chen, X. , Wu, S. , Liu, Z. and Friml, J. (2016) Environmental and endogenous control of cortical microtubule orientation. Trends Cell biol. 26, 409–419. 26951762 10.1016/j.tcb.2016.02.003

[pbi13292-bib-0003] Fujioka, S. , Yamane, H. , Spray, C.R. , Katsumi, M. , Phinney, B. , Gaskin, P. , Macmillan, J. *et al* (1988) The dominant non‐gibberellin‐responding dwarf mutant (*D8*) of maize accumulates native gibberellins. Proc. Natl Acad. Sci. USA 85, 9031–9035. 16594001 10.1073/pnas.85.23.9031 PMC282656

[pbi13292-bib-0004] Hamada, T. (2014) Microtubule organization and microtubule‐associated proteins in plant cells. Int. Rev. Cell Mol. Biol. 312, 1–52. 25262237 10.1016/B978-0-12-800178-3.00001-4

[pbi13292-bib-0005] Lawit, S.J. , Wych, H.M. , Xu, D. , Kundu, S. and Tomes, D.T. (2010) Maize DELLA proteins *dwarf plant8* and *dwarf plant9* as modulators of plant development. Plant Cell Physiol. 51, 1854–1868. 20937610 10.1093/pcp/pcq153

[pbi13292-bib-0006] Lee, Y.J. , Hiwatashi, Y. , Hotta, T. , Xie, T. , Doonan, J.H. and Liu, B. (2017) The mitotic function of augmin is dependent on its microtubule‐associated protein subunit EDE1 in *Arabidopsis thaliana* . Curr. Biol. 27(24), 3891–3897.e4. 29225022 10.1016/j.cub.2017.11.030

[pbi13292-bib-0007] Peng, J.R. , Richards, D.E. , Hartley, N.M. , Murphy, G.P. , Devos, K.M. , Flintham, J.E. , James, B. *et al* (1999) ‘Green revolution' genes encode mutant gibberellin response modulators. Nature 400, 256–261. 10421366 10.1038/22307

[pbi13292-bib-0008] Pignocchi, C. , Minns, G.E. , Nesi, N. , Koumproglou, R. , Kitsios, G. , Benning, C. , Lloyd, C.W. *et al* (2009) ENDOSPERM DEFECTIVE1 is a novel microtubule‐associated protein essential for seed development in Arabidopsis. Plant Cell. 21, 90–105. 19151224 10.1105/tpc.108.061812 PMC2648083

[pbi13292-bib-0009] Sasaki, A. , Ashikari, M. , Ueguchi‐Tanaka, M. , Itoh, H. , Nishimura, A. , Swapan, D. , Ishiyama, K. *et al* (2002) A mutant gibberellin‐synthesis gene in rice. Nature 416, 701–702. 11961544 10.1038/416701a

[pbi13292-bib-0010] Winkler, R.G. and Helentjaris, T. (1995) The maize *Dwarf3* gene encodes a cytochrome P450‐mediated early step in Gibberellin biosynthesis. Plant Cell. 7, 1307–1317. 7549486 10.1105/tpc.7.8.1307 PMC160953

